# Metabolic Complications and Increased Cardiovascular Risks as a Result of Androgen Deprivation Therapy in Men with Prostate Cancer

**DOI:** 10.1155/2011/391576

**Published:** 2011-08-01

**Authors:** Bhavin R. Shastri, Subhashini Yaturu

**Affiliations:** ^1^Department of Medicine, Section of Endocrinology and Metabolism, Overton Brooks VA Medical Center, Shreveport, LA 71101, USA; ^2^Department of Medicine, Section of Endocrinology and Metabolism, 113 Holland Avenue, Stratton VA Medical Center, Albany, NY 12208, USA

## Abstract

Prostate cancer is one of the most common malignancies in men. Charles Huggins and Clarence V. Hodges reported the androgen dependence of prostate cancer in 1941. That led to the utilization of androgen deprivation therapy as an important therapeutic modality to treat prostate cancer. Androgen deprivation therapy has additional systemic effects that include sexual dysfunction, psychological changes and more important are the metabolic changes. Metabolic changes in particular include insulin resistance, increase fat mass and low-density lipoprotein cholesterol, and induce type 2 diabetes. In this review we will focus on the cardiovascular risk associated with androgen deprivation therapy that includes the mechanisms involved.

## 1. Introduction

Prostate cancer (PC) is one of the most common malignancies in men and the second most common cause of mortality among cancers in men after lung cancer. In recent years, incidence of PC has been rising, probably due to new and effective screening guidelines. The Centers for Disease Control (CDC) reports that in 2007 [1], (the most recent year numbers are available) 223,307 men in the United States were diagnosed with prostate cancer and 29,093 men in the United States died from prostate cancer. Per the report of the National Cancer Institute, 2,311,000 have prostate cancer and the numbers projected for 2020 will be 3,266,000 [2]. Beside watchful waiting, standard treatments for PC are surgery, radiation therapy, and hormone therapy. Androgen dependence of PC was first described by Huggins et al. in 1941 [3]. Depletion of gonadal testosterone through androgen deprivation therapy (ADT) is the frontline treatment for advanced prostate cancer and may be accomplished by medical or surgical castration. In the current era, ADT is the primary therapy for localized disease, as an adjunct to radiation therapy for high-risk localized disease and as treatment for biochemical relapse (prostate-specific antigen (PSA) rise only) after failure of localized therapy, often with uncertain benefits [4, 5]. The modalities of ADT include orchiectomy, radiation, and medical castration (antiandrogens, LHRH agonists LHRH antagonists), with latter being the most commonly used. Continuous pituitary stimulation by GnRH agonists overcomes endogenous pulsatile GnRH and suppresses LH release, resulting in low serum testosterone, rather acting as LHRH antagonists. Besides its benefit, ADT is known to cause several endocrine complications such as osteoporosis, decreased libido, changes in cognition and mood, hot flushes, and gynecomastia [6]. In recent years, insulin resistance, diabetes, dyslipidemia, adverse body composition, and metabolic syndrome emerged as metabolic effects of ADT, which are contributing to increased cardiovascular mortality in this population [7–12]. The purpose of the paper is to familiarize the reader with clinical evidence on potential metabolic complications of ADT and provide a perspective to make educated decisions about the survival benefit.

## 2. Evidence Acquisition

We performed MEDLINE searches (1950–2010) of English literature using terms: androgen deprivation therapy, insulin resistance, metabolic syndrome, diabetes, dyslipidemia, adverse body composition, and hyperglycemia. The bibliography of the literature found was reviewed for additional relevant articles which were included in the review.

## 3. Body Mass Index (BMI), Insulin Resistance, and Prostate Cancer

Epidemiologic studies have shown that higher serum levels of insulin and insulin resistance are associated with an increased risk of PC, even after adjustment for BMI, body fat, levels of sex hormone, and insulin-like growth factor 1 (IGF-1) [13]. Because insulin is a known growth factor, there is a possibility that insulin even may be responsible for the stimulation of PC cells. Furthermore, insulin may increase the risk of PC by stimulating IGF-1 synthesis [14], which is known to stimulate growth of the prostate.

## 4. ADT and Prostate Cancer

PC continues to be a major health issue in the USA. One in 6 men will develop prostate cancer at some point in his life. Incidence of PC has been increasing probably secondary to the increased early detection rate with wider use of PSA testing. Prostate cancer is largely an androgen-sensitive disease. ADT is conventionally used for patients with advanced disease. For localized disease, traditional therapies were watchful waiting, external beam radiation therapy, and radical prostatectomy. Studies have shown that androgen deprivation may delay tumor progression and improve survival after radical therapy [15, 16]. Two main forms of ADT are medication (antiandrogens, LHRH agonist) or surgical (orchiectomy), the former being much more common [17] (Table 4). Medical ADT is achieved with a luteinizing hormone-releasing hormone agonist such as leuprolide (Lupron) or goserelin (Zoladex), or an antiandrogen drug such as flutamide or bicalutamide (Casodex), or a combination of each [18]. ADT is now prescribed to men without evidence of metastatic disease before beginning radical therapy; men with prostate-specific antigen (PSA) relapse after local therapy even in the absence of clinical or radiographic evidence of metastatic disease, and for men with evidence of metastatic disease. ADT is also used as adjunct therapy for men undergoing radiation therapy for high-risk localized disease. With the increase in the number of prostate cancer cases seen in the United States, the use of ADT as a form of treatment has continued to rise. Cancer of the Prostate Strategic Urologic Endeavor (CaPSURE), which is a national disease registry of men with prostate cancer, was screened to identify patients who received treatment with primary ADT (PADT) between 1989 and 2002 for clinically localized disease (T1-T3, Nx/N0, Mx/M0) [19]. This study noted that 14.1% with clinically localized disease received primary ADT. In a similar study, the dominant forms of hormone therapy were luteinizing hormone-releasing hormone monotherapy (48.6%) and combined androgen blockade (LHRH agonist and antiandrogens) (38.8%). At 5 years after the initiation of primary ADT, 67.3% of patients still were receiving treatments with only androgen deprivation [19]. Substantial variation exists in terms of primary treatment selection for localized prostate cancer [20]. ADT adjuvant to radiation therapy increases survival in men with intermediate, high-risk, and locally advanced disease [21–23]. Furthermore, there is a survival benefit for men treated with ADT after radical prostatectomy who also have lymph node involvement [15]. Morote et al. [24] established a direct relationship between testosterone increases and androgen-independent progression. In a group of 73 men with prostate cancer treated with 3 months of depot LHRH agonist, they found that breakthrough responses, with increases of 20–50 ng/dL, occurred in 31%, while breakthrough responses of >50 ng/dL occurred in 25% of patients. Breakthrough increases in testosterone greater than this threshold predicted a lower survival free of androgen-independent progression [24]. 

## 5. Adverse Effects of ADT

The effects of ADT include not only suppression of tumor growth, but also adverse effects on various bodily functions and male health (Table 1). Besides sexual dysfunction and vasomotor symptoms, ADT is known to cause osteoporosis, anemia, gynecomastia, depression, and cognitive decline. Also, it has been noticed recently that ADT increases the incidence of the metabolic syndrome, insulin resistance, and cardiovascular risk [32]. Normal serum testosterone levels are required for normal virilization in men. Low testosterone is associated with decreased libido and energy. Decreasing serum testosterone can have a significant negative impact on quality of life for patients treated with ADT. Although erectile dysfunction is not uncommon after radical prostatectomy, men who undergo ADT have a further decline in ability for sexual intercourse and a decrease in sexual desire compared with men who are not treated with ADT [33]. 

It has been well documented in the literature that male hypogonadism is known to cause several endocrine complications such as osteoporosis, sexual dysfunction, changes in cognition and mood, hot flushes, gynecomastia, decreased lean body and muscle strength, and quality of life [6]. Testosterone also plays a major role in muscle strength and bone density. It has been also shown that ADT has a significant effect on walking speed and physical performance in men with prostate cancer [34]. Patients treated with ADT experienced more symptoms, have worse fatigue [35], loss of energy, emotional distress, and a lower overall quality of life than men who deferred hormone therapy. Also it has been shown that combined androgen blockade had a greater adverse effect on quality of life than monotherapy [36]. However, these effects are well documented in the literature and are not reviewed in detail here. In recent years, the focus has turned towards metabolic side effects of ADT and its role in increasing cardiovascular mortality in this group of patients. 

## 6. Testosterone and Metabolic Effects

Testosterone has several desirable and undesirable effects on body metabolism. Nishiyama et al. showed that after 6 months of ADT, body weight, levels of fasting blood sugar, serum total cholesterol, blood urea nitrogen, compensated calcium, inorganic phosphorus, bone-specific alkaline phosphatase, and compensated urinary deoxypyridinoline increased significantly. Peripheral red blood cell counts, hemoglobin level, hematocrit, uric acid, and radial bone density decreased significantly [37]. It has been also shown in a small trial that hypogonadism in males with prostate cancer results in a rise in the augmentation of central arterial pressure, suggesting large artery stiffening. Adverse body compositional changes associated with rising insulin concentrations suggest reduced insulin sensitivity. These adverse hemodynamic and metabolic effects may increase cardiovascular risk in this patient group [25]. Testosterone plays a significant role in metabolism of adipose tissue stores. It also has a role in direct regulation of vascular tone and vascular compliance through both endothelial-dependent and -independent mechanisms. Testosterone plays a crucial role in body composition, regulating lean body mass, insulin concentration, and sensitivity. Testosterone is directly involved in mobilization of free fatty acids. Data suggest that testosterone is negatively related to total cholesterol, low-density lipoprotein, and triglyceride levels but positively related to serum high-density lipoprotein. These beneficial effects are thought to be mediated through its aromatization to estrogen. Testosterone replacement in men results in an improvement in insulin sensitivity by increasing glucose disposal in the muscle [38]. Furthermore, testosterone administration in men results in hyperplasia of Type 1 skeletal muscle fibers, which are responsible for glucose uptake [39, 40].

## 7. Lipid Peroxidation and Antioxidant Systems in Prostate Cancer and Effect of Antiandrogen Therapy

Oxidative damage occurs as a result of deficient antioxidant defensive mechanisms due to the effect of endogenous and exogenous factors. In a study to evaluate the effect of prostate cancer and antiandrogenic therapy on lipid peroxidation and antioxidant systems, Iynem and associates [41] evaluated malondialdehyde level (MDA) as an indicator of lipid peroxidation, erythrocyte glutathione (GSH) level as an indicator of antioxidant state, glutathione reductase (GSH-Red), glutathione peroxidase (GSH-Px), and glutathione-S-transferase (GST) as antioxidant enzymes and plasma vitamin E level. They noted that erythrocyte GSH levels, the activities of GSH-Red and GSH-Px, and plasma vitamin E levels were found to be significantly low (*P* < 0.01, *P* < 0.05, *P* ≤ 0.001, and *P* ≤ 0.001 resp.), whereas plasma MDA levels and erythrocyte GST activity were higher in patients with prostate cancer when compared with the healthy subjects. With antiandrogen therapy, there was a significant decrease in plasma MDA levels and significant increase in erythrocyte GST activity in the patient group [41], indicating a shift in the oxidant-antioxidant balance towards the oxidative state in patients with metastatic prostate cancer. Similar findings were noted by Surapneni and Ramana [42]. 

## 8. ADT and Hyperglycemia, Insulin Resistance, and Diabetes

There is a significant negative correlation between total and free testosterone levels and levels of fasting glucose, insulin, leptin, and HOMA-IR (Table 2). Long-term ADT patients are at risk for developing insulin resistance and hyperglycemia, thus leading to their increased risk of cardiovascular disease. Testosterone deprivation was shown to cause increased insulin concentration despite unchanged plasma glucose, which is suggestive of insulin resistance. This adverse metabolic profile developed independent of age and BMI and appeared to be a direct result of androgen deprivation [12]. A short-term prospective study of 22 men with PC undergoing ADT showed a significant increase in insulin levels after 3 months of treatment compared with baseline; however, there was no significant change in plasma glucose levels [25]. Smith et al. reported that ADT significantly increased fasting plasma insulin by 26% and decreased insulin sensitivity by 13% [28]. Another short-term study showed that ADT for 3 months resulted in a 63% increase in fasting insulin levels without any changes in fasting glucose [26]. These observations suggest that insulin resistance (manifested by hyperinsulinemia) develops within a few months of starting ADT; however, this hyperinsulinemia is sufficient to prevent the development of hyperglycemia. It has been now also shown that men on long-term ADT (at least 12 months) were not only insulin resistant but also had developed frank hyperglycemia [12]. Keating et al. in her observational study (population-based cohort of 73,196) showed GnRH agonist use was associated with increased risk of incident diabetes with HR of 1.44 and CAD with HR 1.16. It is also associated with MI with HR of 1.11 and sudden cardiac death with HR 1.16 [10]. Men treated with orchiectomy were more likely to develop diabetes but not coronary heart disease, myocardial infarction, or sudden cardiac death in the same study [10]. Basaria et al. showed that men taking ADT had higher levels of leptin even after adjusting it for BMI and there was a negative correlation between testosterone levels and leptin levels. This indicates that low testosterone levels were responsible directly for high leptin levels [12]. Furthermore, a recent longitudinal study showed that the elevation in leptin levels with aging is a direct consequence of decline in testosterone levels and not because of changes in BMI [43]. Similarly, high leptin levels in men have been associated with an increased risk of PC. Leptin may promote the growth and survival of PC cells by several mechanisms. Leptin upregulates the signaling of signal transducer and activator of transcription 3, which has an antiapoptotic role and is important for the growth and survival of PC cells. Furthermore, it has been shown that leptin promotes angiogenesis, which plays a crucial role in PC metastases. This finding is supported by the fact that high-grade PC lesions on pathology show strong immunoreactivity for leptin receptor [12].

## 9. ADT and Immune System and Inflammatory Markers

Animal studies have shown that castration results in an inflammatory state [44]. These adrenal glucocorticoid and immune over responses observed in gonadectomized mice were shown to be reversed by testosterone treatment [44]. In a study of men with hypogonadism (Klinefelter syndrome) with autoimmune disorders, testosterone replacement has improved immunological parameters [45]. These data support the notion that testosterone may influence immune cell activation and prevent autoimmune disease development. Using Cox proportional hazard models, C-reactive protein (CRP) association with prostate cancer, no association was noted by one group [46], and higher CRP is associated with shorter survival [47] by a different group of scientists, suggesting inconsistent results [46, 47]. The recent report by Smith et al showed in his 12 week prospective trial that ADT is associated with decrease in insulin sensitivity index and increase in adiponectin levels, but resistin, CRP and plasminogen activator inhibitor type-1 did not changed significantly [48]. It also causes reduced vascular compliance resulting from impaired endothelial release of mediators such as nitric oxide contributes to arterial stiffening. However, data are not universal on this matter. Combined androgen blockade with leuprolide and bicalutamide significantly increased serum adiponectin levels by 37.4% ± 7.2% from baseline to week 12 (*P* < 0.001) but did not alter the resistin, CRP, or PAI-1 levels [48]. Interleukin-6 (IL-6) and CRP were reported to be elevated in prostate cancer patients and IL-6 may potentially be involved in the development or progression of prostate cancer [49]. Adiponectin levels were reported lower in PC than in controls [50]. In a prospective study, CRP appeared unrelated to prostate cancer risk [51]. In a recent study to assess the tumoural presence and cellular location of CRP using tissue microarray technology, Elsberger et al. noted CRP presence in the cytoplasm and nucleus of selected tumours [52]. Cytoplasmic CRP correlation positively with metastases at diagnosis (*P* = 0.039), whereas nuclear CRP presence correlation with metastases at relapse (*P* = 0.006). A trend towards an increase in cytoplasmic and nuclear CRP presence from hormone sensitive to hormone refractory tumours was noticed [52]. Since tumoural CRP is likely to have a role in progression of prostate cancer, it was suggested by authors that CRP is associated with increased presence of metastases at the time of diagnosis and time of relapse [52]. From the Apolipoprotein MOrtality RISk (AMORIS) study with repeated measurements of CRP in a prospective cohort study noted a positive trend between CRP and risk of developing prostate cancer and concluded a link between inflammatory markers and cancer risk [53].

## 10. ADT and Dyslipidemia

The potential detrimental effects of dyslipidemia in cardiovascular disease have been well appreciated over many years. Recent studies have demonstrated that low testosterone concentrations are associated with lower levels of high density lipoprotein cholesterol and higher triglyceride, total cholesterol and low density lipoprotein concentrations [53]. This association persists even after adjusting for BMI. Different prospective and retrospective studies (Table 3), however, showed slightly different results. It has been shown consistently in various studies that ADT increase total cholesterol level. Some studies also showed increases in triglyceride level [28, 54, 55] versus others, which did not show significant elevations in triglyceride level in the ADT group [25, 26, 53]. Although total cholesterol was often increased during ADT therapy, HDL-C also found to increase in some studies, which might decrease the adverse consequence of increased total cholesterol. Most of the prospective trials fail to show increases in LDL-C level, although a recent cross-sectional study showed that men on long term ADT had significantly higher LDL cholesterol compared with the control group [53]. Yannucci et al demonstrate that statistically significant increases in total cholesterol, triglyceride, LDL-C and HDL-C can occur during the first 6 months of ADT. Interestingly these changes were observed even in patients on statin therapy [54]. Another study showed orchiectomy caused hypercholesterolemia and an increase in both total and LDL apolipoprotein B. In that study, the high density lipoprotein concentration was not affected despite a reduction in plasma testosterone [56]. Chen et al studied changes in plasma cholesterols, lipoproteins, and apolipoproteins (Apo) B-100, A-I, and A-II profiles over 2.5 years. They found significant decreases of HDL-C, Apo A-I and Apo A-II and an increase of triglyceride levels and VLDL associated with ADT [55]. In the same study, Apo B-100 levels were not statistically different between ADT and control group. Another long prospective study in 40 men undergoing ADT for 48 weeks showed increases in all total cholesterol, low density lipoprotein, high density lipoprotein and triglyceride levels [29]. On other hand, one short prospective study of 3 months duration did not show any change in total cholesterol, LDL-C, HDL-C or triglyceride level in contrast to most of the studies [25]. 

## 11. ADT and Body Composition

In healthy males, testosterone levels are inversely related to the degree of central abdominal obesity (Table 1). In men with low testosterone, improvement in visceral adiposity and muscle mass has been reported with testosterone replacement therapy. The mechanism for effect of testosterone on body fat metabolism is not very well defined though one hypothesis is that androgen receptors are known to be present on visceral adipocytes. It is likely that testosterone is directly involved in the mobilization of free fatty acids. ADT causes a decrease in lean body mass and an increase in fat body mass consistently [57]. Literature differs in the area of increased fat, subcutaneous versus abdominal fat. It has been shown that PC patients have higher body weight, a higher percentage of body fat, and are more likely to be obese [30]. Increased fatness resulted primarily from accumulation of subcutaneous tissue rather than intra-abdominal adipose tissue [29]. ADT for prostate cancer results in accumulation of both visceral and subcutaneous abdominal fat. Increased visceral fat area appears more closely linked to testosterone than estradiol deficiency. Increased insulin resistance may arise secondary to visceral fat accumulation, rather than as a direct result of sex steroid deficiency [58]. ADT may indirectly affect glucose and lipid metabolism by mediating changes in body composition, especially increased visceral fat accumulation. However, it has also been hypothesized that testosterone can directly modulate hepatic and lipoprotein lipases in visceral adipose tissue, affecting insulin and lipid levels [59]. It has been shown in various cross-sectional and prospective trials that ADT in men with PC significantly decreases lean body mass and increases fat mass (Table 1). Basaria et al. showed that men with PC undergoing ADT have increased fat mass in both visceral and subcutaneous areas compared with control patients [27]. Another short term (3 months) study also confirmed similar increase in fat mass and a significant reduction in lean body mass [25]. Chen et al. reported long-term followup (1–5 year) in men undergoing ADT therapy and changes in fat mass and obesity prevalence. Unlike most other studies, the decrease in lean body mass was not significant but changes in weight and fat mass were statistically significant [30]. Long-term studies (12 months followup) also showed similar results of decreased lean body mass and increased fat mass in patients undergoing ADT for PC [31, 57]. Galvão et al. also reported lean body mass and fat mass changes similar to others although they also showed that increase in fat accumulation was in all regional sites (upper limb, lower limb, trunk), with greater changes for the limbs than trunk [60]. Most recent study by Smith et al. showed 4.3 ± 1.3% increase in fat mass and 1.4 ± 0.5% decrease in lean body mass associated with men undergoing ADT for a 3-month period [48]. In a study to evaluate the effect of hypogonadism secondary to ADT on the vascular system reported that the changes in fat mass correlate with increased insulin levels over a 3-month period (*r* = 0.56; *P* = 0.013) [25].

## 12. ADT: Cardiovascular Disease and Mortality

Cardiovascular disease is the major cause of death worldwide. It has been shown that premenopausal women have lower risk of cardiovascular disease than postmenopausal women. The protective role of normal estrogen level has been studied widely, however, till recently, limited information was available for androgen deprivation and cardiovascular risk. Several studies have reported an association between ADT and an increased risk of cardiovascular events, including myocardial infarction and cardiovascular mortality [7, 9, 10, 61].

### 12.1. ADT: Potential Mechanisms of Cardiovascular Disease (Figure 1)

Prospective clinical trials have demonstrated that ADT may increase cardiovascular disease risk by increasing body weight, reducing insulin sensitivity, and/or resulting in dyslipidemia [62]. Insulin itself to some extent acts as a vascular hormone and is known to be an important regulator of vascular compliance in large arteries. It is known that due to insulin resistance, ADT causes high insulin levels at equal blood glucose level. As reported above, ADT has been shown to be associated with the loss of lean body mass and increase in adipose tissue, mainly visceral area. These changes in body composition and insulin resistance are associated with the increased risk of cardiovascular disease. Although only moderate increase in risk of diabetes and heart disease in men receiving ADT, it could have substantial negative impact on the health of old age, frail prostate cancer survivors, especially given the considerable increases in its use among the men with local prostate cancer. It has been shown that approximately half of men with PC die of causes unrelated to the cancer itself, with CVD being the most common noncancerous etiology. An earlier report had shown that, after the deaths directly attributable to PC and its complications, CVD was the second leading cause of death (responsible for 27% of the deaths) [63]. However, a recent report showed that non-PC-related deaths now exceed PC-related mortality, with CVD being the single most common cause of non-PC-related deaths [64]. Although prostate cancer specific mortality is decreasing, there is little effect on overall mortality in this population, suggesting the possibility of an increased risk of death from nonprostate cancer-related causes. Newly diagnosed prostate cancer patients who received ADT for at least 1 year were found to have a 20% higher risk of serious cardiovascular morbidity compared with similar men who did not receive ADT [9]. Efstathiou et al. have shown in a large randomized trial of men treated with radiotherapy and ADT that greater baseline BMI is independently associated with higher prostate cancer specific mortality in men with locally advanced prostate cancer. However, it is unclear whether weight loss after prostate cancer diagnosis alters the survival or not [65]. On other hand, evidence also points toward obesity as an independent risk factor for mortality and prostate cancer metastasis regardless of ADT [66]. Beer et al. characterize changes in hemoglobin (Hb) levels after the initiation of ADT in patients with previously untreated, metastatic prostate cancer. Overall, 3 months after initiating ADT, the mean Hb level declined 0.54 g/dL. After adjusting for potential confounders, including baseline Hb level, a decline in Hb after 3 months of ADT was associated independently with shorter survival (hazards ratio [HR], 1.10 per 1 g/dL decline; *P* = 0.0035) and shorter progression-free survival (HR, 1.08 per 1 g/dL decline; *P* = 0.013) [67] In another study, after a period of 2.5 years on ADT, four patients out of twenty-four were found to be affected by coronary heart disease. In that study, ischemic coronary arteriosclerosis with an incidence rate of 16.6% as caused by prolonged ADT is mediated through changes in HDL cholesterol, Apo A-I and Apo A-II profiles [55].

## 13. Conclusion

Prostate cancer is one of the leading causes of death in men, but despite this, a substantial proportion of patients with prostate cancer die of other unrelated causes. Comorbid conditions are common in this group, but a particularly strong association has been noted between the presence of cardiovascular disease and the eventual cause of death. This raises the possibility that prostate cancer itself or the treatment used in some way aggravates the natural course of vascular disease. Decisions about ADT in PC should be taken after weighing the benefit of cancer-specific outcome versus potential increase in risks of metabolic complication and cardiovascular disease. Future research is needed to define the situation for which benefits of ADT outweigh the risks. Because of substantial evidence, it is clear that physicians need to discuss the potential cardiovascular and metabolic risk of this therapy before starting the treatment as the overall survival benefit is not as evident. Future research is also required to identify populations at higher risk of adverse effect of ADT and for specific prevention strategies in this group. Also, it is critically important to determine the role of routine prevention measures like diet, exercise, and lipid-lowering agents in reducing potential cardiovascular risks. Until further evidence in prospective trials is available, careful selection of patients, selective screening of risk factors, focused efforts to reduce cardiac risks, and improvement in modifiable metabolic risk factors may mitigate some of the adverse effects of ADT.

## Figures and Tables

**Figure 1:**
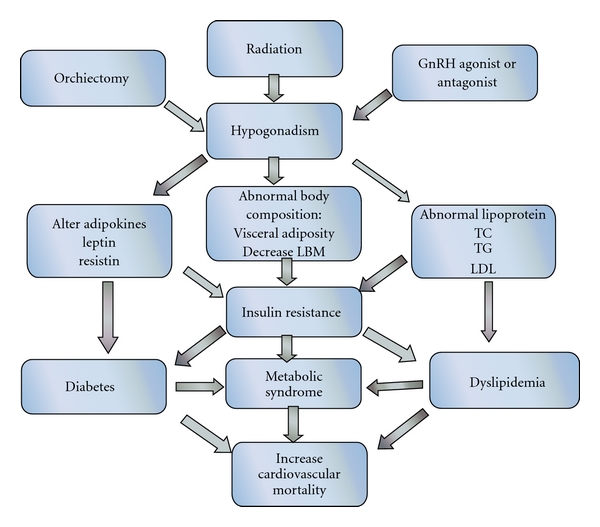
Mechanisms that lead to diabetes and cardiovascular risk with prostate cancer therapy.

**Table 1:** Effects of ADT.

(1) Sexual dysfunction
(2) Decreased libido
(3) Impotence
(4) Decreased lean body mass
(5) Increased fat mass
(6) Decreased quality of life
(7) Gynecomastia
(8) Hot flushes
(9) Insulin resistance
(10) Metabolic syndrome
(11) Increased TC
(12) Increased TG
(13) Increased LDL
(14) Changes in cognition
(15) Hyperglycemia
(16) Increased blood urea nitrogen
(17) Decreased red blood cell counts
(18) Decreased hemoglobin
(19) Decreased hematocrit
(20) Decreased uric acid
(21) Increased arterial pressure
(22) Increased arterial stiffness
(23) Increased leptin
(24) Increased resistin
(25) Increased cardiovascular mortality

**Table 2:** Effects of ADT on glucose metabolism.

	Duration	HOMA	Hyperglycemia	Insulin level
Smith et al. [25]	3 months	NR	NS	Increased 63% with ADT (*P* = 0.02)
Dockery et al. [26]	3 months	NR	NS	Increased 63%*
Basaria et al. [27]	12–101 months	Higher with ADT*	Higher in ADT*	Higher with ADT*
Smith et al. [28]	12 weeks	NR	NS	Higher with ADT*

NS: Not significant; NR: Not reported; *P < 0.001.

**Table 3:** Effects of ADT on lipid metabolism.

	Duration	TC	TG	LDL	HDL
Smith et al. [25]	3 months	NS	NS	NS	NS
Dockery et al. [26]	3 months	⇑	NS	NS	⇑
Smith et al. [28]	12 weeks	⇑	⇑	NS	⇑
Braga-Basaria et al. [11]	12–101 months	⇑	NS	⇑	NS

NS: Nonsignificant; ⇑: Increased; TC: Total Cholesterol; TG: Triglycerides; LDL: Low density lipoprotein Cholesterol; HDL: High-density lipoprotein Cholesterol.

**Table 4:** Effect of ADT on body composition.

	Duration	Lean body mass	Abdominal area	BMI/Weight	Fat mass
Basaria et al. [27]		NS	NR	Higher in ADT group*	Higher at total body, trunk and extremities
Smith et al. [25]	3 months	Decreased 1.7 kg, *P* = 0.016	NR	NR	Increased 1.7 Kg
Smith et al. [29]	4 months	Decreased 2.7%*	Increased by 3.9 + 1.2%, *P* = 0.003	Increased by 2.4 + 0.8%	Increased by 9.4 + 1.7%*
Chen et al. [30]	1–5 yr	NS	NR	Higher in ADT group*	Higher in ADT
Lee et al. [31]	12 Months	Decreased by 2%*	NR	NR	Increased by 6.6%*

NS: Not significant; NR: Not reported  *P < 0.001.
